# Enolase-1 and Inflammation

**DOI:** 10.3390/biom16081156

**Published:** 2026-08-08

**Authors:** Rafael Fernandez, Asha Jacob, Monowar Aziz, Ping Wang

**Affiliations:** 1 Center for Immunology and Inflammation, The Feinstein Institutes for Medical Research, 350 Community Drive, Manhasset, NY 11030, USA; rfernandez10@northwell.edu (R.F.); avarghes@northwell.edu (A.J.); maziz1@northwell.edu (M.A.); 2 Department of Surgery, Donald and Barbara Zucker School of Medicine at Hofstra/Northwell, 350 Community Drive, Manhasset, NY 11030, USA

**Keywords:** Enolase-1, inflammation, sepsis, ischemia/reperfusion, macrophages, ENOblock

## Abstract

Enolase-1 (ENO-1) is classically known as a highly conserved glycolytic enzyme that catalyzes the conversion of 2-phosphoglycerate to phosphoenolpyruvate in the final steps of glycolysis. This enzyme, however, is being increasingly implicated as a multifunctional moonlighting protein with compartment-specific roles in inflammation. Within the cytosol, ENO-1 regulates macrophage inflammation during sepsis; on the cell surface, it functions as a plasminogen receptor, and extracellularly, it can participate in innate immune signaling. Across innate and adaptive immunity, ENO-1 has been implicated in macrophage activation, neutrophil recruitment, endothelial cell dysfunction, fibroblast remodeling, and autoantigenicity. These functions have been linked to sepsis, acute respiratory distress syndrome, acute organ injury, hemorrhagic shock, rheumatoid arthritis, and cancer-associated inflammation in the tumor microenvironment. Therapeutic targeting of ENO-1 includes small-molecule inhibitors and monoclonal antibodies. ENO-1, with its compartment-specific functions in disease pathogenesis, serves as a significant therapeutic target for inflammatory diseases. In this review, we discuss the novel compartment-specific roles of ENO-1 in inflammatory diseases, defining its functions beyond its role in glycolysis. We conclude that both the metabolic and moonlighting functions of ENO-1 contribute to inflammation, and future studies should delineate its compartment-specific roles in inflammatory pathophysiology, as compartment-specific targeting may represent the future of ENO-1-directed therapy.

## 1. Introduction

Inflammation is the body’s immune response triggered by harmful stimuli and conditions, which is coordinated by a wide range of mediators that create complex regulatory networks. Briefly, the acute inflammatory response involves the organized movement of blood components (plasma and leukocytes) to the site of injury or infection [1]. The recruitment of inflammatory cells from the blood, along with tissue-resident immune cells, provides a rapid way to eliminate pathogens and contribute to healing [2]. The initial insult is detected by tissue macrophages or mast cells via pattern recognition receptors, which then activate proinflammatory cytokines, chemokines, and vasoactive amines to further amplify the immune response. These mediators increase vascular permeability and recruit circulating neutrophils, which then transmigrate through the endothelium [3]. While a localized immune response can be beneficial, a dysregulated or inappropriate inflammatory response can be catastrophic [4]. Cells involved in the pro-inflammatory response must rapidly provide energy to fuel inflammation, which is accomplished by glycolysis and high lactate production [2].

Glycolysis is a highly conserved metabolic process that is responsible for the anaerobic catabolism of glucose and the production of adenosine triphosphate (ATP). The glycolysis pathway consists of 10 metabolic enzymes that convert one molecule of glucose into two pyruvate molecules. This results in the net gain of two molecules of ATP, two molecules of reduced nicotinamide adenine dinucleotide (NADH), and two molecules of water per molecule of glucose. Under physiologic oxygenated conditions, the pyruvate generated is transported to the mitochondria, where it undergoes oxygen dependent oxidation to form acetyl coenzyme A (acetyl co-A). This is then metabolized by the tricarboxylic acid cycle and the electron transport chain during oxidative phosphorylation (OXPHOS) to produce 36 molecules of ATP from one molecule of glucose. In the cells lacking mitochondria (e.g., erythrocytes) or in cells experiencing hypoxia, glycolysis becomes the primary source of ATP production. Under these conditions, there is an increase in glycolytic flux which is essential to maintain ATP production and homeostasis [5].

In various pathological states, such as in oncogenesis or infection, cellular metabolism is altered and glycolysis instead of OXPHOS becomes the main source to metabolize glucose. This is known as the Warburg effect [6]. Although glycolysis yields less ATP per molecule of glucose than OXPHOS, it provides metabolic intermediates that support the biosynthetic requirements of rapidly proliferating cells. Thus, cells with a higher rate of metabolism, despite lower ATP production, may gain a selective advantage when competing for limited energy sources [7]. During peak inflammation for instance, immune cells primarily rely on glycolysis for energy to produce metabolic intermediates required by other biosynthetic pathways essential for cellular growth and differentiation. The levels of these metabolites and glycolytic enzymes inside the cells can then influence the activation and suppression of signaling pathways and the post-transcriptional regulation of inflammatory genes. Cells involved in the proinflammatory response, such as M1 macrophages, must provide rapid energy to fuel inflammation, which is accomplished through glycolysis. However, immune cells during the resolution phase rely mainly on OXPHOS for metabolism [2].

During the inflammatory response, glycolytic metabolic intermediates and enzymes are essential for cellular growth and differentiation, and the cellular levels of these metabolites and enzymes determine the activation and suppression of signaling pathways, the epigenetic and post transcriptional regulation of inflammatory genes, and the post translational modifications of proteins. Phosphoenolpyruvate (PEP), succinate, citrate, itaconate, alpha-ketoglutarate, lactate, and 2-hydroxyglutarate have been shown to have an impact on the inflammatory states of cells [2]. Of the numerous metabolites and glycolytic enzymes that are unbalanced during the glycolytic inflammatory response, enolase-1 (ENO-1) is emerging as particularly interesting and clinically relevant. Enolase (EC 4.2.1.11) [8] is a metalloenzyme that catalyzes the conversion of 2-phosphoglycerate (2PG) to PEP in glycolysis. This glycolytic enzyme has been reported to exhibit altered expression in several disease pathologies and has been detected to drive diverse processes, including plasminogen binding, maintenance of mitochondria membrane stability, RNA chaperone activity, and signal transduction [9]. Although it is expressed in most cells, the gene that encodes *ENO-1* is not just a housekeeping gene, but also exhibits expression variations under different pathophysiologic, metabolic, and developmental conditions. For instance, *ENO-1* mRNA is significantly upregulated during cellular growth and is nearly undetectable during the quiescent phase [10]. This review focuses on ENO-1 function as a moonlighting protein across different cellular compartments and its distinct roles in inflammation. It also examines inflammatory diseases in which ENO-1 has been linked to disease pathophysiology or has been investigated as a therapeutic target, with an emphasis on sepsis and shock, autoimmune disease, and cancer-associated inflammation. The above diseases were selected based on evidence directly linking ENO-1 to inflammatory mechanisms or therapeutics. To avoid overgeneralization, the findings are discussed according to cellular compartment, immune cell type and disease context.

## 2. Enolases

**Figure 1:**
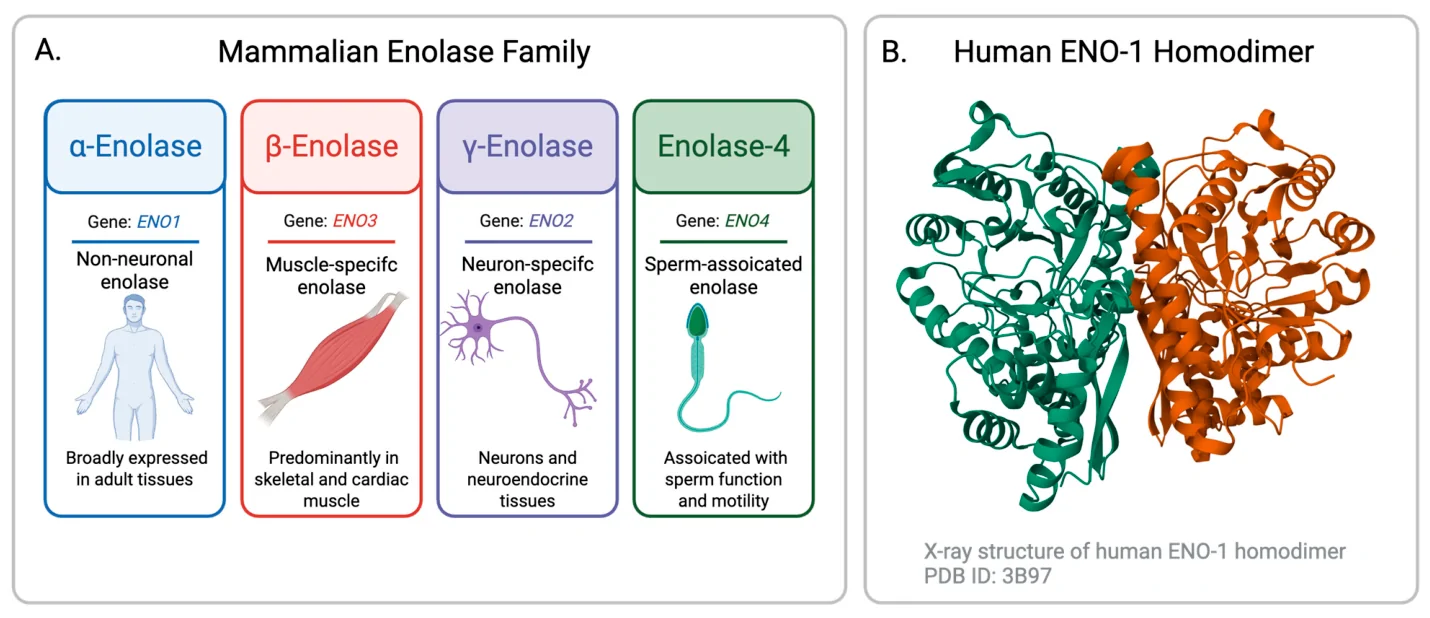
Mammalian enolase family and structural organization of human ENO-1. (**A**) Overview of the four mammalian enolase family members. α-Enolase (ENO-1; encoded by *ENO1*) is broadly expressed in adult tissues; β-enolase (ENO-3; *ENO3*) is predominantly expressed in skeletal and cardiac muscle; γ-Enolase (ENO-2; *ENO2*) is primarily expressed in neurons and neuroendocrine tissues; and enolase-4 (ENO4; *ENO4*) is a spermatogenic cell-specific enolase-related protein associated with sperm structure and motility. (**B**) Ribbon representation of the X-ray crystal structure of the human ENO-1 homodimer, with the two monomers shown in green and orange (Protein Data Bank ID: 3B97) [19]. Created in BioRender. Fernandez, R. (2026) https://BioRender.com/h25qrl9 (accessed on 22 July 2026).

Enolase was first characterized in muscle extract in 1934 by Lohmann and Meyerhof, with future studies showing that there are four enolase isoenzymes that exist in mammals (Figure 1): α-enolase (ENO-1), which is present in almost all mature tissues; β-enolase (ENO-3), which exists primarily in muscle tissues; and γ-enolase (ENO-2), which is mainly found in the nervous and neuroendocrine tissues [11]. Enolase is the key glycolytic enzyme that catalyzes the dehydration of 2PG to PEP, a reaction that occurs in the final steps of glycolysis. Enolase is a metalloenzyme, meaning it requires the metal ion magnesium (Mg^2+^) to be catalytically active. It is a highly conserved enzyme and exists in a dimeric form (homo- or heterodimer), composed of two subunits arranged in an antiparallel fashion [10]. During development, the expression of these genes changes significantly. *α-enolase (αα)* can be replaced by muscle specific *β-enolase (ββ or αβ)* or neuron-specific *γ-enolase (γγ and αγ)* [12]. ENO-2 is primarily found in neurons as the cells of the neuroendocrine system, where it plays a role in the pathophysiology of the nervous system, with diverse functions as a neurotrophic-like factor promoting neuronal growth, differentiation, and survival, and as a biomarker for neuroinflammation-induced neurodegeneration [13]. ENO-3 is a muscle-specific enolase isoform that also catalyzes the conversion of 2PG to PEP but is predominantly expressed in adult skeletal muscle and is associated with muscle development and regeneration [14]. Enolase 4 (ENO4) is a spermatogenic cell-specific enolase-related protein associated with sperm structure and motility. In mice, ENO4 has been found to contribute to fibrous sheath assembly and sperm motility, and disruption of *ENO4* results in impaired sperm motility and male infertility [15].

ENO-1 is also known as 2-phospho-D-glycerate hydrolase, and it is a highly conserved enzyme that is abundantly expressed in most cells. While it is classically known for its role in glycolysis as a metalloenzyme that catalyzes the conversion of 2-phosphoglyceric acid to phsophoenolpyruvic acid, ENO-1 is a multifunctional enzyme involved in cellular stress, bacterial and fungal infections, the metastasis of cancer, and the growth and development of organisms [11]. While abundantly present in the cytoplasm, the significance of ENO-1 depends heavily on its subcellular location. ENO-1 can move to the cell surface, where it can act as a plasminogen-binding receptor, assist in the recruitment of inflammatory cells, or can be secreted as a soluble protein [9]. By using a different start codon, the *ENO-1* gene can also give rise to a *myc* promoter binding protein-1 (MBP-1), a 37–38 kDa protein that binds to the *c-myc* P2 promoter to negatively regulate the transcription of the protooncogene, making ENO-1 a potential candidate for tumor suppression [16]. *ENO-1* specific mRNA is consistently reported to be increased at very high levels in exponentially growing cells, but remains almost undetectable in the resting and quiescent cellular phases [17]. Thus, ENO-1 is not simply acting as a metabolic enzyme but is a versatile moonlighting protein that is at the intersection of immunometabolism, cellular stress responses, and pathophysiology. Moonlighting proteins are defined as single-polypeptide chains that perform multiple functions that are not explained by gene fusion, alternative splicing, or secondary catalytic activities [18]. Full-length ENO-1 meets this definition because the same protein acts as a cytosolic enzyme, cell-surface plasminogen receptor, and an extracellular inflammatory mediator with different functions depending on its compartmentalization [9]. This classification provides an understanding of how ENO-1 can produce distinctive inflammatory effects based on its subcellular location.

## 3. Compartmentalization of ENO-1

### 3.1. Cytosolic ENO-1

**Figure 2:**
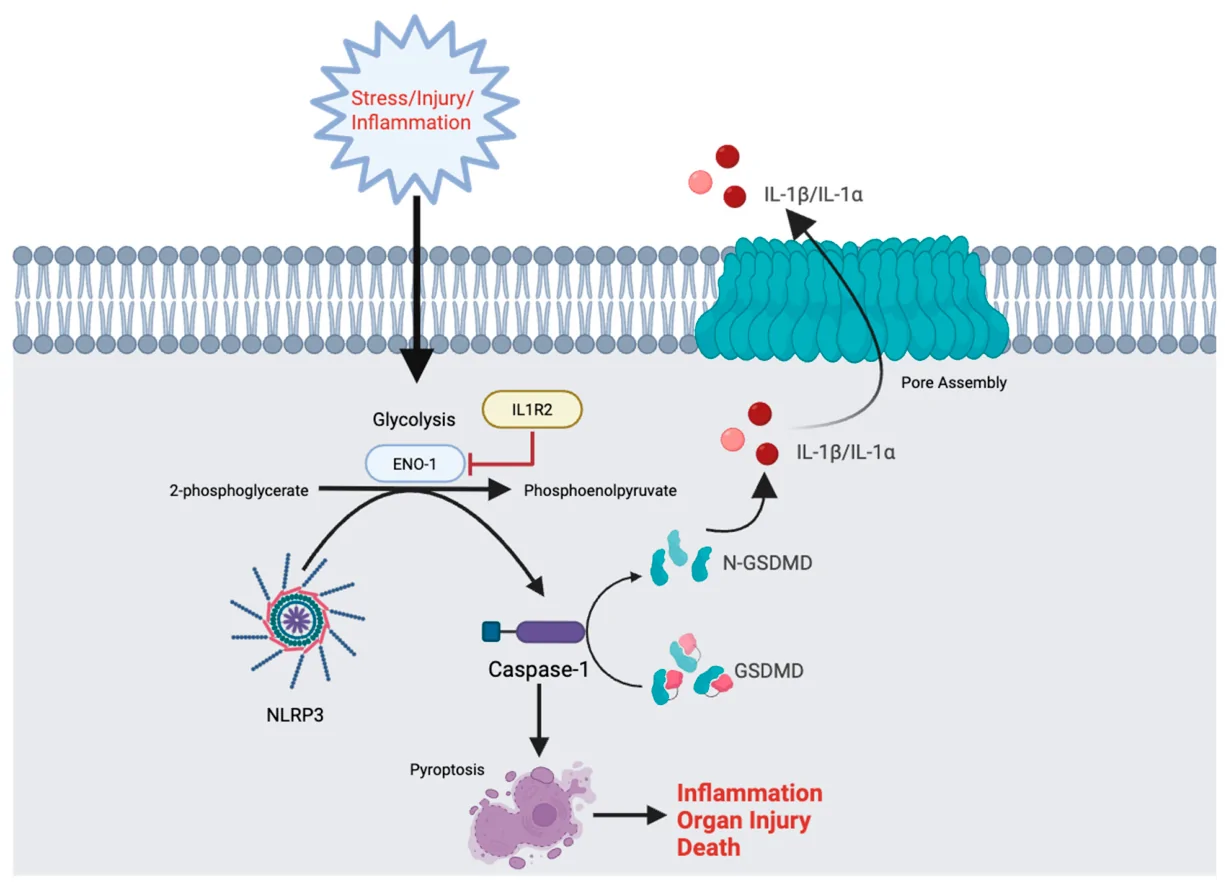
Cytosolic ENO-1 links glycolysis to macrophage pyroptosis and inflammatory injury in sepsis. ENO-1 catalyzes the conversion of 2-phosphoglycerate to phosphoenolpyruvate in glycolysis. IL1R2 interacts with ENO-1 to suppress its enzymatic activity and limit glycolysis. ENO-1-dependent glycolysis promotes caspase-1 activation and cleavage of gasdermin D. N-terminal gasdermin D then oligomerizes to the plasma membrane to facilitate the release of inflammatory cytokines and induce pyroptosis to amplify organ injury in sepsis. Created in BioRender. Fernandez, R. (2026) https://BioRender.com/ipwfk9c (accessed on 23 July 2026). Abbreviations: ENO-1, enolase-1; GSDMD, gasdermin D; IL-1α, interleukin-1 alpha; IL-1β, interleukin-1 beta; IL1R2, interleukin-1 receptor type 2; N-GSDMD, N-terminal gasdermin D; NLRP3, NOD-, LRR-, and pyrin domain-containing protein 3.

Within the cytosol, ENO-1 functions as part of the glycolytic response that supports inflammatory activation and plays a critical role in the metabolic reprogramming in immune cells. During hyperinflammatory responses, there is a shift in immune cell metabolism toward aerobic glycolysis, which produces the metabolic intermediates needed for other biosynthetic pathways necessary for cellular growth and differentiation [2]. Recent work supports the role of cytosolic ENO-1 as a regulator of inflammatory cell metabolism rather than a constitutively expressed glycolytic enzyme. Interleukin-1 receptor type 2 (IL1R2), for instance, acts as a competitive inhibitor of IL-1-mediated signaling to dampen inflammatory responses in immune cells. Proteomic screening and co-immunoprecipitation have revealed a strong interaction between IL1R2 and ENO-1, with IL1R2 suppressing ENO-1 mediated glycolysis in macrophages. Upon stimulation with lipopolysaccharide (LPS), glycolysis increased by 28.2% and 62.4% in wild-type and *IL1R2^−/−^* macrophages respectively, using a Seahorse extracellular flux analyzer to monitor the extracellular acidification rate, a measure of glycolysis. Mechanistically, IL1R2 suppressed glycolysis-mediated pyroptosis by reducing gasdermin D (GSDMD) expression. Targeting ENO-1 with ENOblock, a small molecular inhibitor of ENO-1, reduced inflammatory cytokine expression, organ injury, and mortality in septic mice [20]. This novel study suggests that ENO-1 is not just a passive glycolytic enzyme, but acts as an active regulator of macrophage inflammation during sepsis, thus linking glycolytic reprogramming to inflammasome-mediated cell death (Figure 2).

ENO-1 has also been linked to other cellular stress responses as well. Exposure of cells or tissues to elevated temperatures induces the synthesis of a family of proteins called heat-shock proteins. Enolase has been identified as the yeast heat shock protein HSP48 [21], as well as a hypoxic stress protein. Oxygen (O_2_) is an essential nutrient that serves as a key substrate in cellular metabolism, and in a large variety of physiological and pathological states, organisms encounter insufficient O_2_ availability, or hypoxia. To cope with this stress, the primary transcriptional response is mediated by hypoxia-inducible factors (HIFs). The response to hypoxic stress is closely linked to the immune response through nuclear factor-kappa B (NF-κB) signaling, with several studies demonstrating how endothelial cells react to hypoxia and how HIFs facilitate this response [22]. In cultured vascular endothelial cells, five cell-associated stress proteins (Mr 34, 36, 39, 47, and 57), also known as hypoxia-associated proteins, are upregulated when exposed to hypoxia. The 47 kDa hypoxia-associated protein has been identified as non-neuronal ENO-1, which is significantly up-regulated in endothelial cells exposed to hypoxia, suggesting that ENO-1 upregulation might contribute to hypoxia tolerance by increasing anaerobic energy glycolytic capacity in endothelial cells [23]. These findings support a role for ENO-1 as a stress responsive intracellular protein whose expression and function are altered under various conditions such as hypoxia or heat stress.

### 3.2. Cell Surface ENO-1

**Figure 3:**
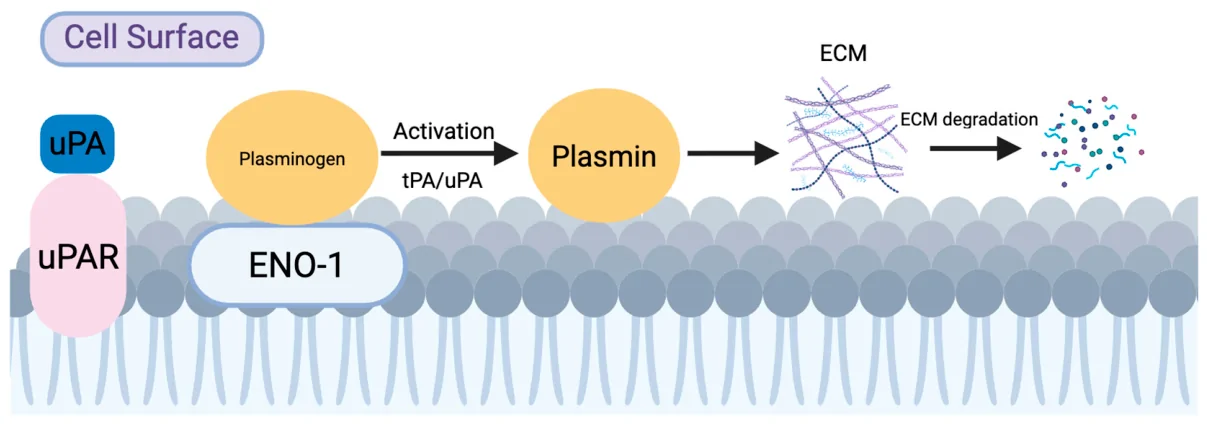
Cell surface ENO-1 links plasminogen activation to extracellular matrix degradation. Cell-surface ENO-1 functions as a plasminogen receptor by binding to plasminogen on the plasma membrane. uPAR localizes uPA to the cell surface. In the presence of tPA or uPA, plasminogen is converted to plasmin. Plasmin then contributes to pericellular proteolysis and extracellular matrix degradation. Created in BioRender. Fernandez, R. (2026) https://BioRender.com/u4qzlvi (accessed on 26 July 2026). Abbreviations: ENO-1, enolase-1; tPA, tissue-type plasminogen activator; uPA, urokinase-type plasminogen activator; uPAR, urokinase-type plasminogen activator receptor.

Although ENO-1 is traditionally recognized as a cytosolic glycolytic enzyme, there is accumulating evidence that indicates that ENO-1 can re-localize to the cell surface or enter the extracellular space, where it participates in immunologic functions. ENO-1 was first identified as a cell-surface plasminogen receptor on U937 cells, a human monocytic cell line [24], and further work expanded on this, showing that ENO-1 is expressed on the surface of monocytes and neutrophils where it binds plasminogen and promotes its activation in a profibrinolytic role [25]. Using U937 monocytic cells as a model and an ENO-1-specific monoclonal antibody 9C12, researchers identified the surface-exposed epitope of ENO-1, which spans 16 amino acids (spanning 257–272, 257-DLDFKSPDDPSRYISP-272) [26]. Another proposed ENO-1 plasminogen motif suggests that the ENO-1-plasminogen interaction is mediated by the binding of plasminogen kringle domains to the C-terminal residues K420, K422, and K434 of ENO-1 [24]. Since then, ENO-1 has been found to be expressed on the cell surface of many different hematopoietic cells, such as monocytes, T cells, and B cells, as well as neuronal and endothelial cells, where it acts as a plasminogen receptor to promote cell migration in various pathophysiological states, including the inflammatory response [27]. Plasminogen is an inactive circulating enzyme that is produced mainly by the liver. When plasminogen binds to fibrin, or cell surface receptors such as ENO-1, it can be converted by tissue plasminogen activator (tPA) or urokinase-type plasminogen activator (uPA) into plasmin, an active serine protease. Although plasmin is primarily known for degrading fibrin during fibrinolysis, it can also degrade components of the extracellular matrix, thereby remodeling the pericellular environment. This process enables the trafficking of inflammatory cell and tissue remodeling [28,29]. To examine the role of ENO-1 in the pericellular generation of plasmin activity, Lopez-Alemany et al. created a monoclonal antibody (mAB), mAb11G1, that blocked plasminogen binding to purified ENO-1. They found that this antibody blocked 90% of the cell-dependent plasminogen activation by either tPA or uPA across multiple leukocyte cell lines and in peripheral blood neutrophils and monocytes. They also demonstrated that α-enolase is present in both the cytosolic and plasma membrane fractions, with roughly 6% of the total ENO-1 detected in the plasma fraction. This suggests that while surface ENO-1 is only a minor fraction of total cellular ENO-1, it is a key enzyme in promoting plasminogen activation and pericellular plasmin generation (Figure 3) [30].

In acute inflammation, ENO-1 is upregulated on the neutrophil surface, where it can also promote plasminogen dependent neutrophil migration and neutrophil extracellular trap (NET)-associated tissue injury, whereas anti-ENO-1 blockade reduce neutrophil recruitment in LPS-induced lung injury, supporting ENO-1 as a key extracellular regulatory of neutrophil inflammation [31]. LPS was also found to translocate ENO-1 from the cytosol to the monocyte cell surface without altering total *ENO-1* mRNA or protein expression. This translocation increased plasminogen-dependent pericellular proteolysis and enhanced monocyte migration and transmigration. ENO-1 overexpression also boosted monocyte recruitment to the acutely inflamed lung in vivo. Additionally, patients with pneumonia showed increased surface ENO-1 on peripheral blood monocytes and ENO-1 staining in alveolar mononuclear cells, supporting the role of surface ENO-1 in inflammatory leukocyte trafficking [32].

The molecular mechanisms underlying ENO-1 translocation and its transport across the cell membrane remain largely unknown, but they may involve nonclassical trafficking. Zakrzewicz et al. demonstrated that ENO-1 localizes to caveolae, interacts with caveolin-1, and annexin A2. They also found that depleting either *caveolin-1* or *annexin A2* decreases surface ENO-1 without affecting overall ENO-1 levels [33]. Didiasova et al. demonstrated that inflammatory stimulation of the metastatic breast cancer cell line MDA-MB-231 with LPS enhances ENO-1 exteriorization through a Ca^2+^-dependent, Stromal interaction molecule 1 (STIM1)/Calcium release-activated calcium channel protein 1 (ORAI1)-mediated pathway, which not only promotes ENO-1 accumulation on the cell surface but also its release into the extracellular space in exosomes [34]. Additional studies have shown a relationship between caveolin-1 and Ca^2+^ signaling. A study in breast cancer cells had shown that caveolin-1 overexpression increased store-operated Ca^2+^ entry, whereas caveolin-1 knockdown reduced it [35]. Separately, calcium signaling was shown to induce caveolin-1 transcription [36]. Based on these findings, we can speculate that elevated intracellular Ca^2+^ may facilitate the transcription of *caveolin-1*. Caveolin-1 may then enhance store-operated Ca^2+^ entry via STIM1/ORAI1-mediated pathway causing ENO1 translocation to the cell surface in caveolae, interacts with caveolin 1 and annexin A2. Future studies are, however, needed to determine how ENO1 is exteriorized.

### 3.3. Extracellular ENO-1

Beyond its cytosolic and cell surface functions, ENO-1 can also be detected extracellularly, where it participates in innate immune signaling. In rheumatoid arthritis (RA), ENO-1 is not only an intracellular metabolic enzyme but also an extracellular inflammatory autoantigen that builds up in the joint space. Synovial fluid from RA patients contains citrullinated proteins, and ENO-1 levels are higher in RA patients than in osteoarthritis controls. Both native and citrullinated ENO-1 serve as antigens in the rheumatoid joint, with antibodies to citrullinated enolase-1 peptide 1 (CEP-1) detected in a significant number of RA synovial fluid samples [37]. An earlier study from the same group reached similar conclusions, showing that ENO-1 is abundant in the synovium of RA patients, with high levels of antibodies against citrullinated ENO-1 than against native ENO-1, further supporting ENO-1’s role in the inflamed joint [38]. Beyond its role as an autoantigen, extracellular ENO-1 has immunostimulatory activity. In vitro, soluble ENO-1 cultured with monocytes induces the production of pro-inflammatory cytokines TNF-α, IL-1β, and IL-6, and ENO-1-treated monocytes produce higher levels of chemokines such as CCL3, CXCL1, and IL1. This occurs through a CD14-dependent Toll-like receptor 4 (TLR4) signaling pathway, indicating that ENO-1 can act as a pro-inflammatory ligand [39]. Complementing this, cell-surface ENO-1 was found to be expressed on mononuclear cells derived from RA patients. When peripheral blood mononuclear cells (PBMCs) were stimulated with an anti-ENO-1 antibody, they produced inflammatory cytokines, including TNF-α, IL-1α/β, IL-18, and Interferon-gamma (IFN-γ) via the p38 mitogen-activated protein kinase (MAPK) and NF-κB pathways [40]. Together, these findings show that extracellular ENO-1 can enhance RA pathogenesis as both a soluble damage-associated molecular pattern (DAMP) and as a surface-expressed autoantigen targeted by RA autoantibodies.

Outside of the joint, soluble ENO-1 has been associated with tissue injury and leukocyte trafficking. In kidney stones, calcium oxalate crystals increase ENO-1 secretion from renal tubular cells. ENO-1 promotes crystal and monocyte invasion through the renal interstitium, suggesting a paracrine role for ENO-1 in renal inflammation [41]. In acute traumatic injury, ENO-1 levels were found to be elevated in the plasma of blunt trauma patients compared to healthy controls. The same study showed that ENO-1 activated pulmonary endothelial cells, increased intercellular adhesion molecule-1 (ICAM-1) expression, and primed neutrophils through a plasmin and protease-activated receptor 2 (PAR-2) dependent mechanism [42]. Overall, these studies support that extracellular ENO-1 can act as an active mediator of inflammation to increase leukocyte recruitment and tissue damage across different disease states.

Considered together, ENO-1’s cytosolic, cell-surface, and extracellular roles in inflammation are best viewed as likely parallel and possibly concurrent. In sepsis, for example, macrophage cytosolic ENO-1-dependent glycolysis promotes caspase-1 activation, GSDMD cleavage, cytokine, and pyroptosis [20]. In parallel, LPS was found to induce rapid translocation of ENO-1 from the monocyte cytosol to the cell surface, where it promotes plasminogen-dependent pericellular proteolysis and leukocyte migration [32]. Soluble extracellular ENO-1 may provide an additional inflammatory pathway by activating CD14-dependent TLR4 signaling and cytokine production [39] and promoting endothelial cell activation [42]. Whether one pathway is upstream of the other, or whether they act together, has not yet been established and may depend on the cell type and the disease of interest. Future studies should selectively inhibit compartment-specific ENO-1 to determine if these pathways occur together, independently, simultaneously, or sequentially.

### 3.4. Nuclear ENO-1

In the nucleus, an ENO-1 derived product plays a regulatory role beyond its canonical glycolytic function, acting as a transcriptional repressor. Feo et al. showed that in addition to the full-length 48 kDa ENO-1 protein, the ENO-1 transcript can derive a shorter product generated from an internal AUG at Met97. This truncated 37 kDa product localizes to the nucleus, binds to the *c-myc* P2 promoter, and represses *c-myc* promoter activity, supporting its role as a *Myc* promoter-binding protein-1 (MBP-1), and a transcriptional regulator of the *c-myc* protooncogene. The *c-myc* protooncogene is a critical regulator of cell proliferation, differentiation, and apoptosis [16]. Beyond its role in tumor cells, *c-myc* may also influence inflammatory stromal hyperplasia and myeloid cell phenotypes. In rheumatoid arthritis synovial fibroblasts, *c-myc* inhibition reduced cartilage invasion, suggesting that *c-myc* repression could decrease fibroblast invasiveness [43]. Tumor-conditioned media was also found to induce macrophage *c-myc*, and inhibition of *c-myc* reduced the expression of VEGF, MMP9, HIF-1α, and TGF-β, suggesting that repression of *c-myc* could limit tumor-associated inflammation [44]. While ENO-1/MBP-1 were not examined in either study, we speculate that MBP1-mediated repression could limit fibroblast invasiveness and tumor-associated inflammation. Future studies are needed for confirmation.

While the best-characterized nuclear function of ENO-1 is repression of *c-myc*, reported MBP-1 targets include c-myc, *cyclooxygenase-2 (COX-2)*, *and ERBB2* [45]. COX2 is a key enzyme in prostaglandin synthesis, contributing to inflammation, pain, and fever [46]. Thus, while the inflammatory role of nuclear ENO-1 is less well established than that of cytosolic or cell surface ENO-1, future work should explore the link between the truncated form of ENO-1 and its role in inhibiting inflammatory transcription by repressing genes such as *COX-2*.

## 4. ENO-1 in Innate Immunity

### 4.1. Macrophages

Macrophages are one example of how ENO-1 acts as a link between immunometabolism and inflammation, where it functions not only as a glycolytic enzyme but also participates directly in inflammatory responses. Recent proteomic screening has identified ENO-1 as a binding partner of IL1R2 in macrophages. IL1R2 acts as a decoy receptor to limit cytokine activity and dampen inflammatory responses in immune cells. IL1R2 was found to suppress ENO-1 activity, thereby inhibiting glycolysis, reducing gasdermin D-mediated pyroptosis, and attenuating inflammation in macrophages. The same study then examined the effects of the ENO-1 inhibitor, ENOblock, in a murine cecal ligation and puncture (CLP)-induced sepsis model and found that treatment with ENOblock significantly lowered plasma aspartate aminotransferase (AST) and alanine aminotransferase (ALT) levels, plasma lactate, and proinflammatory cytokine expression, and improved survival [20], supporting cytosolic ENO-1 as a regulator of macrophage inflammatory biology.

ENO-1 has also been found to contribute to the recruitment of macrophages and monocytes. Cell-surface-associated proteolysis plays a role in the migration of mononuclear phagocytes to the sites of inflammation. ENO-1 is known to bind plasminogen at the cell surface to induce plasmin production. Wygrecka et al. have shown that LPS can rapidly upregulate ENO-1 cell-surface expression on monocytes via translocation from cytosolic pools, thereby increasing plasmin production and enhancing monocyte migration through epithelial monolayers. In vivo, intratracheal LPS promoted the recruitment of monocytes that overexpressed ENO-1 to the alveolar compartment of the acutely inflamed lung, and in patients with pneumonia were found to have increased surface ENO-1 on circulating monocytes and increased ENO-1 on mononuclear cells in the alveolar compartment. These findings suggest that cell-surface ENO-1 promotes the recruitment of monocytes/macrophages to inflamed tissues [32].

Beyond migration, ENO-1 has been shown to activate monocytes and macrophages as a soluble inflammatory ligand. Guillou et al. have shown that ENO-1 binds to monocytes and induces pro-inflammatory cytokine secretions via a CD14-dependent TLR4 pathway [39]. Complementing this, Bae et al. have shown that cell-surface expression of ENO-1 is increased on monocytes and macrophages from rheumatoid arthritis patients, and that antibodies against ENO-1 can stimulate these cells to produce proinflammatory cytokines such as TNF-α, IL-1α/β, IFN-γ, via p38 MAPK and the NF-κB pathway [40]. Taken together, these findings suggest that ENO-1 can regulate macrophage and monocyte biology at several different levels, including cytosolic inflammatory metabolism, recruitment, and cytokine activation.

### 4.2. Neutrophils

Neutrophils are another innate immune cell in which ENO-1 has an important inflammatory effect. Surface ENO-1 expression on neutrophils has been found to be upregulated after LPS stimulation in vitro and in vivo. The anti-ENO-1 mAb 7E5 was also found to disrupt the ENO-1 plasminogen interaction to inhibit neutrophil invasion. In LPS-challenged mice, a marked influx of neutrophils into the alveolar space was observed, and treatment with 7E5 significantly reduced neutrophil infiltration, proinflammatory cytokine levels, and NETosis in LPS-induced acute lung injury (ALI) [31]. These findings suggest that neutrophil surface ENO-1 is not only a marker of activation but can also regulate neutrophil recruitment and tissue injury.

### 4.3. Endothelial Cells

In endothelial cells, ENO-1 is not only a glycolytic enzyme, but it also appears to function as a stress-responsive mediator. ENO-1 is a hypoxia-associated stress protein in endothelial cells. Aaronson et al. identified a 47 kDa hypoxia-associated protein (HAP47) in cultured vascular endothelial cells subjected to hypoxia and found it to be ENO-1. The ENO-1 protein was mainly increased in the cytoplasmic fraction, suggesting that ENO-1 overexpression may help endothelial cells tolerate hypoxia by enhancing anaerobic metabolic capacity [23]. More recent studies suggest that ENO-1 contributes to inflammatory and vascular remodeling programs. In pulmonary endothelial cells, extracellular ENO-1 can directly activate pulmonary microvascular endothelial cells and prime neutrophils. ENO-1 was also found to be markedly increased in the plasma from blunt trauma patients who later developed organ failure, with an increase of 10.8-fold compared to healthy controls. In vitro, cultured human pulmonary microvascular endothelial cells treated with ENO-1 increased ICAM-1 surface expression, promoted neutrophil adherence, and co-precipitated with plasmin/plasminogen and PAR-2. These findings suggest that extracellular ENO-1 may be acting as a pro-inflammatory mediator following trauma, thus leading to endothelial cell activation that amplified leukocyte recruitment and contributes to acute lung injury through a plasmin dependent PAR-2 pathway [42].

A similar pro-inflammatory role for endothelial ENO-1 has been described in lung fibrosis. In primary human umbilical vein endothelial cells (HUVECs), an ENO-1 blocking antibody, HL217, was found to reduce bleomycin-induced plasmin activation and chemokine secretion. Endothelial cells release chemoattractants in response to fibrotic stimuli and plasmin, which may help recruit immune cells to the lungs. HUVECs treated with bleomycin had an increase in surface ENO-1 expression, but not total ENO-1 expression, and plasmin activation could be dose dependently reduced by HL217. Bleomycin also induced the secretion of monocyte recruiting chemokine CCL2 and neutrophil recruiting chemokine IL-8, both of which were reduced by HL217 in a dose-dependent manner [47]. Endothelial ENO-1 has also been implicated in pulmonary hypertension. ENO-1 expression is increased in human pulmonary artery endothelial cells exposed to hypoxia, as well as in lung tissues collected from patients with chronic obstructive lung disease-associated pulmonary hypertension. In vitro inhibition of ENO-1 restored hypoxia induced endothelial dysfunction, including excessive proliferation, angiogenesis, and adhesion. This same study further showed that ENO-1 contributes to mitochondrial dysfunction and activated the Phosphoinositide 3-kinase-protein kinase B-mechanistic target of rapamycin (PI3K-Akt-mTOR) signaling pathways, whereas inhibition of ENO-1 improves pulmonary hypertension and right ventricular dysfunction in vivo [48]. Together, these findings show that ENO-1 plays a critical role in endothelial hypoxic stress, inflammation, and vascular remodeling.

### 4.4. Fibroblasts and Stromal Cells

Compared with other cell types, ENO-1’s role in fibroblasts is less extensive, but evidence indicates that it contributes to fibroblast and stromal cell pathophysiology. *ENO-1* gene and protein expression are upregulated in rheumatoid arthritis fibroblast-like synoviocytes (RA-FLSs) under hypoxic conditions, and inhibition of *ENO-1* by siENO-1 causes a significant decline in RA-FLS proliferation, decreases Bcl-2, survivin, and cyclin B1, and promotes apoptosis. These findings suggest that ENO-1 may contribute to rheumatoid synovial hyperplasia by supporting FLS proliferation and inhibiting apoptosis. While this study showed an ENO-1-dependent proliferative phenotype, siENO-1 was used to reduce total cellular *ENO-1*, and investigators did not determine if this was mediated by cytosolic ENO-1 dependent glycolysis, cell surface ENO-1, or a combination of both [49]. Other studies have shown that FLS survival depends on glycolysis and that TNF can induce TAK1-HIF-1α-GLUT1 glycolytic shift and a proinflammatory phenotype; however, this study did not establish ENO-1 as mediator [50]. Additional evidence suggests that inflammatory stimuli such as TNF-α can promote the translocation of cytosolic ENO-1 to the surface of RA-FLSs and enhance cellular migration [51]. Thus, the current evidence suggests that ENO-1 is important in FLS survival and migration, but they do not examine the compartment-specific functions. Future studies are needed using glycolytic flux and compartment selective ENO-1 inhibition to better delineate the underlying mechanisms.

Beyond arthritis, ENO-1 has been implicated in profibrotic stromal remodeling in idiopathic pulmonary fibrosis (IPF). IPF is an irreversible lung disease secondary to progressive scarring and stiffness of the lungs, resulting in impaired gas exchange, respiratory failure, and death. ENO-1 expression is increased in fibrotic lungs in both patients with IPF and bleomycin-treated mice. In primary human lung fibroblasts, TGF-β increased surface expression of ENO-1 in both normal and IPF-derived cells, and blockade with HL217, an ENO-1-specific antibody, dose-dependently suppressed fibroblast migration and CXCL12-driven chemotaxis. The antibody HL217 also reduced plasmin activation and collagen secretion. In vivo, HL217 lowered inflammatory scores, reduced lung collagen content, and decreased the levels of TGF-β in bronchoalveolar lavage fluid in bleomycin treated mice. Mechanistically, HL217 binds to cell surface ENO-1 where it can inhibit pericellular plasmin activation, thereby limiting migration and inflammatory activity [47]. Taken together, all these findings support a role for surface ENO-1 in promoting fibroblast recruitment and profibrotic activation in lung remodeling.

## 5. ENO-1 in Adaptive Immunity

In addition to its role in innate immunity, ENO-1 is closely tied to the adaptive immunity. Autoantibodies against ENO-1 have been detected in several systemic autoimmune disorders including rheumatoid arthritis [52], systemic lupus erythematosus/lupus nephritis [53], systemic sclerosis [54], Sjögren’s syndrome [55], mixed cryoglobulinemia with nephritis [53], Hashimoto’s encephalopathy [56], inflammatory bowel disease [57], primary biliary cirrhosis [58], Behçet’s disease [59], and autoimmune retinopathy [60]. Autoimmune diseases are a diverse groups of chronic disorders characterized by inappropriate immune responses against self-antigens, resulting in persistent inflammation and tissue destruction [61]. While ENO-1 is unlikely to be the only target of these autoimmune disorders, its recognition by the immune system as a self-antigen suggests it may play a role in disease pathogenesis.

ENO-1 also appears to influence regulatory T cells and has been shown to have a crucial role in modulating the expression of specific splicing forms of Foxp3 and the induction and suppressive function of human Tregs. De Rosa et al. have demonstrated that glycolysis regulates inducible regulatory T-cell generation through ENO-1 nuclear localization. ENO-1 directly repressed Foxp3-E2 expression by binding to *FOXP3* regulatory regions, including the promoter and the conserved noncoding sequence 2 [62]. ENO-1 is also relevant to adaptive immunity in cancer where it acts as a tumor-associated antigen. ENO-1 is overexpressed in pancreatic ductal adenocarcinoma (PDAC) and present on the cell surface of PDAC cell lines, with sera from PDAC patients containing IgG autoantibodies to ENO-1. ENO-1 elicits both a humoral and cellular immune response, including ENO-1 specific antibodies, CD4^+^ and CD8^+^ T cell responses, T cell proliferation, and IFN-γ production to lyse PDAC cells [63]. Niccolai et al. have shown tumor-infiltrating T cells that are specific to ENO-1, which is over-expressed by pancreatic tumors and plays a role in promoting cell migration and cancer metastasis [64]. Overall, ENO-1 is active in adaptive immunity as a self-antigen in chronic inflammatory disease, a regulator of Treg biology, and as a tumor-associated antigen with potential therapeutic relevance.

While ENO-1 has distinct functions in innate and adaptive immunity, these pathways could intersect. In RA, citrullinated ENO-1 can be recognized as a self-antigen [38], while anti-ENO-1 autoantibodies generated through adaptive immunity can bind cell surface ENO-1 on monocytes and promote proinflammatory cytokine production through innate immune signaling via p38 MAPK and NF-kB signaling [40]. 

## 6. ENO-1 in Inflammatory Disease Conditions

### 6.1. Sepsis and Shock

Sepsis and related acute injuries, which are also summarized in Table 1, are among several of the disease states in which the multi-compartmentalized specific functions of ENO-1 come together. In sepsis, ENO-1 appears to support the hyperinflammatory phase by coupling immunometabolism to inflammasome-related injury. Recent work has shown that ENO-1 interacts with IL1R2 in macrophages. Classically, IL1R2 acts as a competitive inhibitor of IL1-mediated signaling, thereby dampening inflammatory responses in immune cells. Proteomic screening and computational modeling found an interaction between IL1R2 and ENO-1, with residues at the IL1R2-ENO-1 interaction interface overlapping with those at ENO-1’s substrate binding site. IL1R2 was found to suppress ENO-1 mediated glycolysis and GSDMD-mediated pyroptosis [20].

Beyond macrophage metabolism, ENO-1 appears to amplify leukocyte recruitment and lung injury through extracellular and cell-surface functions. In monocytes, LPS induces the translocation of ENO-1 from the cytosol to the cell surface without increasing the overall ENO-1 expression. This translocation enhances plasminogen-dependent pericellular proteolysis and directed monocyte migration and transmigration through epithelial cells. In vivo, ENO-1 overexpression increased monocyte recruitment to the acutely inflamed lung, and patients with pneumonia had increased surface ENO-1 on peripheral blood monocytes and ENO-1-positive mononuclear cells in the alveolar compartment [32]. More recently, ENO-1 was identified as a regulator of neutrophil recruitment during acute inflammation. Surface ENO-1 expression on neutrophils was upregulated after LPS stimulation in vitro and in vivo. Antibody-mediated ENO-1 blockage with mAb 7E5 disrupted the interactions between ENO-1 and plasminogen and decreased neutrophil infiltration into the alveolar space in LPS-induced ALI mouse model. The mice with LPS-induced ALI treated with mAb 7E5 also showed a significantly decreased level of proinflammatory cytokines and reduced NETosis [31]. In a murine model of hemorrhagic shock, ENO-1 was upregulated in Kupffer cells in both the in vitro and in vivo setting, and ENOblock treatment reduced liver inflammation, apoptosis, and cleaved caspase-1 expression. This study showed that ENO-1 driven inflammatory response is not solely restricted to sepsis, but that ENO-1 may also participate in sterile inflammation seen after hemorrhagic shock [65].

Extracellular ENO-1 may further propagate acute organ injury by acting directly on the vascular compartment. In blunt trauma patients, circulating ENO-1 levels were elevated compared to healthy controls. ENO-1 was also found to increase ICAM-1 expression, induce polymorphonuclear neutrophil adhesion, and co-precipitate with PAR-2 and plasminogen/plasmin on human pulmonary microvascular endothelial cells. These findings suggest that soluble ENO-1 may act as a circulating inflammatory mediator that induces endothelial activation [42]. Complementing this, soluble ENO-1 has also been shown to activate monocytes through a CD14-dependent TLR4 pathway to induce the production of TNF-α, IL-1β, and IL-6, supporting a DAMP-like function of extracellular ENO-1 in inflammatory states [39]. Transcriptomic analysis showed that ENO-1 is upregulated in sepsis-induced coagulation, and that it is linked to immune regulation pathways, endothelial cell apoptosis, coagulation, and glycosaminoglycan metabolism [66]. Together, these studies show the expanding role of ENO-1 in sepsis, from a mediator of acute inflammatory immunometabolism to a regulator of immune suppression and endothelial injury. The functions of ENO-1 in hyperinflammatory states and immunosuppression may complicate therapeutic targeting, and future anti-ENO-1 therapies may depend on disease stage, cellular compartment, and immune cell type. These findings support the need for temporal-, compartment-, and cell-specific treatments rather than global nonselective ENO-1 inhibition.

### 6.2. Rheumatoid Arthritis and Other Autoimmune Diseases

Rheumatoid arthritis (RA) is a chronic systemic autoimmune disease that primarily affects the lining of synovial joints, with many cellular mechanisms driving the underlying pathophysiology [67]. In RA, ENO-1 is a candidate autoantigen, with antibodies against citrullinated ENO-1 detected in 46% of serum samples from patients with RA, and native ENO-1 is abundantly expressed in the rheumatoid synovium [38]. Subsequent work identified CEP-1 as being highly specific for RA and can cross-react with bacterial enolase, suggesting that post-translational modification of ENO-1 may lead to loss of self-tolerance in RA, with bacterial enolase being a possible environmental trigger [68].

ENO-1 is more than a target in adaptive immunity in RA pathogenesis. Soluble ENO-1 has been shown to bind monocytes in a CD14 dependent TLR4 signaling pathway with ENO-1 being able to induce early production of proinflammatory cytokines such as TNF-α and IL-1β. These cytokines can play a role in RA by perpetuating inflammation and contributing to disease progression in RA. ENO-1 stimulated monocytes were also found to produce multiple chemokines such as CCL3, IL-8, and CXCL1 which are involved in the recruitment and activation of leukocytes [39]. Additional work has shown that antibodies against ENO-1 on monocytes can also stimulate these cells to produce higher amounts of TNF-α, IL-1α/β, and IFN-γ via the p38 MAPK and NF-κB pathways [40]. These findings suggest that ENO-1 can act as both a citrullinated autoantigen and as a surface expressed target of these autoantibodies to produce an inflammatory phenotype. Additional work suggests that ENO-1 may be crucial in the regulation and proliferation of synovial fibroblasts in RA. In fibroblast-like synoviocytes (FLS), *ENO-1* knockout by siRNA decreased cell proliferation, whereas ENO-1 expression was found to promote a significantly higher expression of Bcl-2 and survivin, which promotes cell survival, as well as cyclin B1, a cell-cycle regulator. ENO-1 expression was also found to be associated with reduced cleaved-caspase-3, supporting the role of ENO-1 in the proliferation and survival of synovial fibroblasts [49].

Beyond RA, anti-ENO-1 autoantibodies have also been identified in other autoimmune diseases. In systemic lupus erythematosus, anti-ENO-1 antibodies were detected in 27% of patients, with active renal diseases present in most of the antibody-positive cases. These antibodies were also found in patients with mixed cryoglobulinemia and nephritis but not those without renal involvement, suggesting that anti-ENO-1 autoantibodies were associated with nephritis [53]. In systemic sclerosis, anti-ENO-1 autoantibodies recognized fibroblast-associated ENO-1 and were associated with interstitial lung disease [54]. Autoantibodies targeting the amino-terminal region of ENO-1 were also found to be a potentially useful diagnostic marker for Hashimoto’s encephalopathy [56]. Taken together, these findings show that ENO-1 is recognized as an autoantigen in several autoimmune diseases, and that it may be associated with specific patterns of organ involvement.

### 6.3. Cancer-Associated Inflammation/Tumor Microenvironment

Since ENO-1 is involved in metabolic reprogramming and immune regulation, it is relevant to the inflammatory tumor microenvironment. ENO-1 is expressed on the cell surface as a plasminogen receptor where bound plasminogen is activated to plasmin and contributes to pathological processes such as tumor cell invasion, metastasis, and inflammatory responses. Evidence of the immunologic relevance of ENO-1 in cancer has been particularly well studied in PDAC. Using serological proteome analysis, ENO-1 was identified as a tumor-associated antigen by the host immune system, and patients with PDAC, but not healthy controls, frequently have circulating IgG to ENO-1. ENO-1 was also found to be overexpressed at both the mRNA and protein levels in PDAC tumor tissues compared to normal pancreatic controls. To determine the immunogenic activity of ENO-1, dendritic cells were pulsed with recombinant ENO-1 and cocultured with T-cells. This induced both CD4^+^ and CD8^+^-mediated responses, T-cell proliferation, and IFN-γ production. ENO-1 stimulated T cells also inhibited the growth of PDAC cells in vivo without killing normal cells, suggesting ENO-1 may be a relevant molecular target for immunotherapies in PDAC [63].

Evidence from other tumor types support ENO-1’s role in inflammatory and immune pathways within the tumor microenvironment, with ENO-1 being implicated in the regulation of tumor-associated macrophages. Proteomic analyses of tumor-associated macrophages found that ENO-1 is one of the glycolytic proteins upregulated during macrophage reprogramming when bone marrow-derived macrophages are exposed to tumor extracts [69]. In oral squamous cell carcinoma, ENO-1 promoted tumor cell migration and invasion by modulating IL-6 secretion of macrophages [70]. These findings suggest that ENO-1 may promote tumor progression by contributing to the metabolic and inflammatory functions of macrophages in the tumor microenvironment.

ENO-1 may also be associated with impaired T-cell-mediated antitumor immunity. In bladder cancer, ENO-1 was found to be highly expressed and was associated with CD8^+^ T cell exhaustion, suggesting ENO-1 may contribute to a less effective anti-tumor response [71].

Anti-ENO-1 autoantibodies have also been observed in cancer-associated retinopathy, a progressive, blinding disease that occurs in the presence of systemic tumor growth. Anti-ENO-1 autoantibodies isolated from patients with cancer-associated retinopathy induced retinal cell apoptosis in vitro, supporting a potential role in retinal degeneration [72]. While this response occurs outside the local tumor microenvironment, it shows that immune recognition of ENO-1 in cancer can also contribute to remote inflammatory and autoimmune tissue injury.

Taken together, ENO-1 is also found to be more than a housekeeping glycolytic enzyme in cancer and appears to function as a mediator of tumor associated inflammation. Being compartment specific, intracellular ENO-1 appears to support the Warburg effect [73]; surface ENO-1 promotes plasminogen dependent invasion of cancer cells [74], and as a surface antigen ENO-1 has an impact on adaptive immunity and antitumor responses [63]. ENO-1 may therefore contribute to the tumor microenvironment via its effects on tumor-cell metabolism, macrophage activity, and T cell function.

**Table 1:** Disease-specific roles of ENO-1 and anti-ENO-1 autoantibodies in inflammatory and inflammation-associated conditions.

Disease State	Study Context	Principle Findings	References
*Acute Inflammation, Sepsis, Organ Injury*			
Sepsis	Macrophage pyroptosis during sepsis	ENO-1-dependent glycolysis promoted GSDMD-mediated macrophage pyroptosis and inflammatory injury. Binding of IL1R2 to ENO-1 suppressed its enzymatic activity, glycolysis, and pyroptosis	[20]
	Sepsis-induced coagulopathy	Multi-omics analyses associated ENO-1 with immune regulation, endothelial cell apoptosis, coagulation, and glycosaminoglycan metabolism	[66]
Acute lung inflammation and pneumonia	Monocyte recruitment during pulmonary inflammation	LPS promoted ENO-1 translocation to the monocyte surface, increased plasminogen activation, pericellular proteolysis, and migration. Increased surface ENO-1 was also found on monocyte from patients with pneumonia	[32]
	Neutrophil recruitment during acute lung inflammation	LPS stimulation increased surface ENO-1, whereas antibody blockade of the ENO-1-plasminogen interaction reduced neutrophil migration and NET formation	[31]
Hemorrhagic shock	Kupffer-cell activation and liver injury	ENO-1 expression increased after hemorrhagic shock and hypoxia/reoxygenation. Treatment with ENOblock reduced inflammatory cytokines, caspase-1 activation, and liver injury	[65]
Blunt trauma-associated organ injury	Endothelial and neutrophil activation following trauma	Circulating ENO-1 was elevated following blunt trauma. In vitro, extracellular ENO-1 increased endothelial ICAM-1 expression and neutrophil adhesion, and activated neutrophils via a plasmin-dependent PAR-2 pathway	[42]
*Autoimmune Disease*			
Rheumatoid arthritis	ENO-1 autoantigenicity	Native and citrullinated ENO-1 were identified in rheumatoid synovial tissue and fluid. Antibodies against citrullinated ENO-1 were detected in patients	[38,68]
	Soluble ENO-1-mediated monocyte activation	Soluble ENO-1 stimulated monocyte inflammatory cytokine production through CD14- dependent TLR4 signaling	[39]
	Anti-ENO-1 antibody mediated monocyte activation	Antibody binding to surface ENO-1 on monocytes induced inflammation mediator through p38 MAPK and NF-κB signaling	[40]
	Fibroblast-like synoviocytes proliferation and survival	ENO-1 siRNA knockdown reduced fibroblast-like synoviocytes proliferation and increased apoptosis	[49]
Systemic lupus erythematosus	Autoantibody formation and lupus nephritis	Anti-ENO-1 autoantibodies were detected in SLE and were associated with nephritis	[53]
Systemic sclerosis	Antifibroblast antibody responses	Antifibroblast antibodies recognized ENO-1, and ENO-1 antibody reactivity was associated with interstitial lung disease	[54]
Hashimoto’s encephalopathy	Neural autoantibody response	Proteomic screening identified ENO-1 as a human brain antigen recognized by patient serum	[56]
Other autoimmune disease	Primary Sjögren’s Syndrome	ENO-1 identified as an antigen recognized by autoantibodies from patients with primary Sjögren’s syndrome	[55]
	Mixed cryoglobulinemia	Anti-ENO-1 antibodies were detected and associated with renal involvement	[53]
	Inflammatory bowel disease	Anti-ENO-1 autoantibodies were detected in a substantial proportion of patients with inflammatory bowel disease	[57]
	Primary biliary cholangitis	Anti-ENO-1 autoantibodies were found in patients with primary biliary cholangitis	[58]
	Behçet’s Disease	ENO-1 was identified as a target of circulating anti-endothelial cell antibodies in patients with Behçet’s disease	[59]
	Autoimmune retinopathy	Patient-derived anti-ENO-1 autoantibodies targeted the ganglion-cell and inner nuclear layers of retinal tissue, and induced apoptotic retinal-cell death	[60]
*Cancer*			
Pancreatic ductal adenocarcinoma	Tumor metabolism	ENO-1 supported glycolytic metabolism, and ENO-1 knockdown promoted oxidative phosphorylation and tumor cell growth arrest	[73]
	Tumor invasion and metastasis	Targeting ENO-1 with monoclonal antibodies inhibited plasminogen-dependent invasion and metastatic spread of pancreatic cancer cells	[74]
	Tumor associated ENO-1 antigen	ENO-1 induced tumor-specific CD4^+^ and CD8^+^ T cell responses	[63,64]
Breast Cancer	Tumor-associated macrophage metabolic reprogramming	ENO-1 was found to be increased in tumor extract stimulated macrophages in a mammary tumor model. Similar findings were seen in a human monocyte cell line exposed to breast cancer extracts	[69]
Oral squamous cell carcinoma	Tumor macrophage inflammatory signaling	ENO-1 stimulated macrophage IL-6 secretion, which promoted invasion of tumor cells	[70]
Bladder cancer	Tumor immune microenvironment	ENO-1 overexpression was associated with poor prognosis and CD8^+^ T cell exhaustion	[71]
Cancer associated retinopathy	Paraneoplastic retinal damage	Anti-ENO-1 autoantibodies isolated from affected patients induced retinal cell apoptosis in vitro	[72]

## 7. Therapeutic Strategies

Therapeutic strategies targeting ENO-1 can be considered on two levels: suppression of inflammatory immunometabolism broadly and direct ENO-1 targeted therapies. Since inflammatory cells adopt a Warburg-like phenotype upon activation, characterized by high glycolytic flux, upstream approaches that inhibit glycolysis have emerged as anti-inflammatory strategies. Reviews of immunometabolism in inflammation and sepsis have shown inhibition of aerobic glycolysis through targeting of glycolytic regulators such as HIF-1α, PKM2, PFKFB3, and GLUT1 [75]. Experimental work with glycolysis inhibitors such as 2-deoxyglucose, which is a nonmetabolizable glucose analog that inhibits the glycolytic enzyme hexokinase, has shown that it reduces pro-inflammatory factor expression, inflammatory cell accumulation, lung tissue injury, and lethality in sepsis [76]. These interventions broadly affect glycolytic metabolism and help place ENO-1 within the larger context of inflammatory immunometabolism. The remainder of the section is focused on therapeutic strategies directed toward ENO-1 and its functions.

There are several direct enolase-targeting strategies, including small-molecule inhibition (i.e., ENOblock) and blocking antibodies against cell-surface ENO-1, which are summarized in Table 2. ENOblock (AP-III-a4) was first identified in a phenotypic screen as a triazine-based small molecule that increased glucose uptake and acted as an insulin mimetic in adipocytes, with additional anti-inflammatory effects noted when human aortic endothelial cells treated with AP-III-a4 decreased monocyte adhesion and vascular cell adhesion molecule-1 expression in hyperglycemic conditions [77]. Subsequent studies using affinity chromatography and mass spectrometry showed that AP-III-a4 binds enolase directly, and thus the molecule was renamed ENOblock. In biochemical assays, ENOblock inhibited purified enolase activity in a dose-dependent manner and was reported as the first non-substrate small-molecule enolase inhibitor in biological studies. ENOblock was then subsequently used to probe both glycolytic and nonglycolytic enolase functions. Under hypoxic conditions, ENOblock preferentially induced cancer-cell death, consistent with interfering with the way enolase helps cells adapt to low oxygen conditions. Under normoxia, however, ENOblock inhibited cancer cell migration and invasion, suggesting that it can also modulate nonglycolytic enolase functions, potentially including cell surface-associated activities involved in metastasis and motility. Thus, ENOblock is not simply an inhibitor of glycolytic flux, but is a useful tool for studying both the metabolic and moonlighting biology of ENO-1 [78].

Since then, ENOblock has been examined in a wide range of preclinical disease models. In type 2 diabetic mice, ENOblock improved hyperglycemia and hyperlipidemia, and attenuated tissue fibrosis, apoptosis, and inflammatory marker expression in the liver, kidney, heart, and adipose tissue [79]. In a mouse model of diet induced obesity, ENOblock reduced body weight gain, lowered cumulative food intake, increased fecal lipid content, improved glucose and insulin tolerance, lowered hepatic steatosis and fibrosis, and suppressed inflammatory markers in the liver and hippocampus [80]. In spinal cord injury, ENOblock reduced neuroinflammatory markers, reduced gliosis, improved neuroprotection by upregulation of neurofilament protein, and reduced chondroitin sulfate proteoglycan, which is produced by activated glia and acts to block the regrowth of axons following injury [81]. In inflammatory disease models, such as sepsis, Pharmacological inhibition of ENO-1 with ENOblock reduced liver injury, as evidenced by significantly lower plasma AST and ALT levels, decreased plasma lactate levels, and reduced levels of plasma IL-1β, IL-6, and TNF-α levels in septic mice. Treatment with ENOblock also improved the survival in septic mice. In vitro, ENOblock treated LPS stimulated macrophages showed reduced caspase-1 cleavage, GSDMD cleavage, and cell death compared to that of untreated macrophages. Together, these findings support the idea that cytosolic ENO-1 is an active regulator of macrophage inflammatory metabolism in sepsis [20]. In hemorrhagic shock, ENO-1 was found to be upregulated in Kupffer cells. ENOblock reduced HS-induced liver inflammation by decreasing the inflammatory cytokines TNF-α, IL-6, and IL-1β. ENOblock also lessened liver injury and apoptosis through mediating cleaved caspase-1 expression, a key mediator in NLRP3 inflammasome pyroptosis [65]. In psoriasis, a relapsing autoimmune skin disease characterized by excessive epidermal proliferation and inflammatory cell infiltration, ENO-1 is upregulated in keratinocytes, where it interacts with keratin 17 to sustain glycolysis and proliferation, and ENOblock was found to suppress these responses [82]. Together, these findings show that ENOblock can influence ENO-1 pathways across many different diverse disease states.

Monoclonal antibodies directed against cell-surface ENO-1 also represent an attractive therapeutic strategy to target the extracellular moonlighting functions of ENO-1. Early work showed that the anti-ENO-1 monoclonal antibody 11G1 blocked leukocyte plasminogen activation and inhibited plasmin generation by neutrophils and monocytes [30]. More recent work has shown that the ENO-1-blocking antibody HL217 (original name: HuL001) can bind to cell-surface ENO-1 and inhibit pericellular plasmin activation, subsequent plasmin-mediated migration, and pro-inflammatory cytokine production. In bleomycin-induced lung injury, HL217 reduced collagen deposition in the lungs, reduced TGF-β levels in bronchoalveolar lavage fluid (BALF), and improved the Ashcroft Score, a standardized method for estimating the severity of pulmonary fibrosis. HL217 also reduced the recruitment of myeloid immune cells to the lungs, LPS-induced plasmin activation and cytokine secretion in human PBMCs, bleomycin-induced plasmin activation and chemokine secretion in primary human endothelial cells, and collagen secretion in primary human lung fibroblasts [47].

In oncology, the tumor microenvironment (TME) that is created by tumor cells and tumor stroma can secrete various chemokines and cytokines that drive immune cell infiltration, tumor growth, angiogenesis, and metastasis. In prostate cancer, ENO-1 mAb HuL227 inhibited the migration of prostate cancer cells by blocking the ENO-1/plasmin axis, regulating the TME by suppressing monocyte recruitment, and reducing the secretion of chemokine CCL2 and cytokine TGF-β by osteoclasts [83]. In a genetically engineered mouse model of pancreatic cancer, an ENO-1 DNA vaccination induced both antibody and T cell responses, delayed tumor progression, and prolonged survival. These findings suggest ENO-1 as a molecule that couples cancer cell metabolism to adaptive immunity [84]. Subsequent studies in PDAC showed that targeting ENO-1 with monoclonal antibodies inhibited plasminogen-dependent invasion and metastatic spread of pancreatic cancer cells [74]. Together, these findings suggest that antibodies against cell surface ENO-1 can interrupt ENO-1 dependent plasmin activation, leukocyte migration, and inflammation. Recently, this concept of using monoclonal antibodies to target ENO-1 has entered phase 1 testing in idiopathic pulmonary fibrosis to study the safety, tolerability, pharmacokinetics, and immunogenicity of HuL001, an anti-ENO-1 monoclonal antibody (ClinicalTrials.gov Identifier: NCT04540770) [85].

**Table 2:** ENO-1-directed interventions in preclinical inflammatory disease models.

Diseases/Models	ENO1-Directed Intervention	ENO-1 Compartment and Proposed Mechanism	Major Outcomes	References
*1. Acute Inflammation, Sepsis, and Organ Injury*				
Sepsis; macrophage glycolysis and pyroptosis	ENOblock	Cytosolic ENO-1-dependent macrophage glycolysis driving GSDMD-mediated pyroptosis	Decreased glycolysis-mediated pyroptosis, inflammatory cytokine release, organ injury, and mortality	[20]
Hemorrhagic shock-induced liver injury; Kupffer cell hypoxia/reoxygenation	ENOblock	Proposed intracellular Kupffer-cell ENO-1 activity linked to inflammatory activation and caspase-1 signaling	Reduced IL-1β, TNF-α, IL-6, cleaved caspase-1, and liver injury	[65]
GPR43-deficient sepsis; macrophage M1 polarization and CLP sepsis model	AP-III-a4 (ENOblock)	Proposed cytosolic ENO-1 within the HIF-1α-ENO-1 axis regulating macrophage glycolysis and M1 polarization	Reduced iNOS, TNF-α, IL-6, glycolysis, lung injury, and mortality	[86]
LPS-induced lung injury, acute inflammation, necrotic cell challenge	Anti-ENO-1 monoclonal antibody 7E5	Cell surface ENO-1-plasminogen interaction regulating neutrophil migration and NET formation	Reduced neutrophil recruitment, migration, and NET formation	[31]
*2. Autoimmune and Chronic Inflammatory Disease*				
Experimental autoimmune encephalomyelitis; microglia/macrophage activation	Paeoniflorin; ENOblock/ENO-1 knockdown used for validation	Cytosolic ENO-1 associated M1 polarization of microglia/macrophages	Reduced pro-inflammatory microglia/macrophage polarization and improved EAE clinical severity	[87]
Rheumatoid arthritis	ENO-1-specific siRNA; ENOblock	Cell surface ENO-1 as an inflammatory apoB- binding receptor	Reduced apoB-induced IL-1β, IL-6, TNF-α production	[88]
Rheumatoid arthritis PBMCs	GV1001 peptide	Cell surface ENO-1 mediating p38 MAPK/NF-κB inflammatory signaling	Suppressed ENO-1-induced TNF-α, IL-1β, and IL-6 production	[89]
Bleomycin-induced pulmonary inflammation and fibrosis	Anti-ENO-1 monoclonal antibody HL217	Cell surface ENO-1 as a plasminogen receptor	Reduced pulmonary inflammation, immune cell infiltration, TGF-β in BALF, collagen deposition, and fibrosis	[47]
Psoriasis; keratinocyte proliferation and IMQ-induced psoriasis model	ENOblock/ENO-1 knockdown	Intracellular ENO-1 interacting with keratin 17 to support K17 phosphorylation, glycolysis, and keratinocyte proliferation	Suppressed keratinocyte glycolysis and proliferation, improved psoriasiform skin inflammation	[82]
*3. Metabolic Inflammatory Disease*				
Diet-induced obesity and metabolic inflammation	ENOblock	Nuclear ENO-1 associated transcriptional repression of regulators of lipid metabolism, gluconeogenesis, and inflammation	Reduced body weight and attenuated obesity-associated metabolic inflammation in the liver, adipose tissue, and hippocampus	[80]
Type 2 diabetes model with liver and kidney complications	ENOblock	Nuclear ENO-1 associated transcriptional repression linked to metabolic inflammation, fibrosis, and apoptosis	Reduced hyperglycemia, hyperlipidemia, inflammatory markers, tissue apoptosis, fibrosis, and secondary diabetic organ injury	[79]
*4. Cancer and the Tumor Inflammatory Microenvironment*				
Genetically engineered pancreatic cancer mouse model	ENO-1 DNA Vaccine	ENO-1 as a tumor associated antigen inducing humoral and cellular antitumor immunity	Delayed tumor progression and prolonged survival	[84]
Pancreatic ducal adenocarcinoma	Anti-ENO-1 monoclonal antibodies	Cell surface ENO-1 as plasminogen receptor regulating tumor cell invasion	Inhibited plasminogen-dependent invasion and reduced metastatic spread	[74]
Pancreatic cancer; MDSC infiltration into tumor microenvironment	Anti-ENO-1 antibody	Cell surface ENO-1 on MDSCs regulating adhesion, migration, and tumor infiltration	Reduced MDSC adhesion and invasion, shifted T cell cytokine responses toward IFN-γ/IL-17	[90]
Prostate cancer; bone metastasis	Anti-ENO-1 monoclonal antibody HuL227	Cell surface ENO-1 as a plasminogen receptor regulating tumor microenvironment	Reduced tumor growth and osteoclast activation in the bone, secretion of invasion-related cytokines, and inflammation induced migration and chemotaxis of prostate cancer cells	[83]
Pancreatic cancer	ENO-1 DNA vaccine + PI3Kγ inhibition	ENO-1 as a tumor-associated antigen eliciting humoral and cellular immunity; MDSC targeting via PI3Kγ inhibition	Enhanced B-cell/T-cell antitumor immunity and reduced pancreatic tumor growth	[91]

Notes: This table summarized the preclinical studies investigating ENO-1 as a therapeutic target in inflammatory and inflammation-associated disease models, including small-molecule inhibitors, ENO-1 knockdown, monoclonal antibodies, and vaccination. For each study, the disease context, intervention, ENO-1 compartment and proposed mechanism, major outcome, and reference are provided. Abbreviations: apoB, apolipoprotein B; BALF, bronchoalveolar lavage fluid; CLP, cecal ligation and puncture; EAE, experimental autoimmune encephalomyelitis; ENO-1, enolase-1; GPR43, G-protein-coupled receptor 43; GSDMD, gasdermin D; HIF-1α, hypoxia-inducible factor-1 alpha; IFN- γ, interferon-gamma; IL, interleukin; IL-1β, interleukin-1 beta; IL-6, interleukin-6, IL-17, interleukin-17; IMQ, imiquimod; iNOS, inducible nitric oxide synthase; LPS, lipopolysaccharide; MAPK, mitogen-activated protein kinase; MDSC, myeloid-derived suppressor cell; NET, neutrophil extracellular trap; NF-κB, nuclear factor-kappa B; PBMCs, peripheral blood mononuclear cells; PI3Kγ, phosphoinositide 3-kinase gamma; siRNA, small interfering RNA, TGF-β, transforming growth factor-beta; TME, tumor microenvironment; TNF-α, tumor necrosis factor-alpha; Treg, regulatory T cell.

## 8. Controversies and Knowledge Gaps

Despite the growing interest in and literature on ENO-1 as an inflammatory mediator and therapeutic target, several controversies remain. ENO-1 is a highly compartmentalized moonlighting protein, with its activities strongly depending on its cellular and extracellular location [9]. ENO-1 has been implicated as a glycolytic enzyme, a cell-surface plasminogen receptor, a soluble extracellular inflammatory ligand, and a nuclear regulatory protein, but many studies do not clearly specify which compartment-specific function is targeted. In addition, the mechanisms underlying ENO-1 trafficking to the cell surface or extracellular space remain incompletely understood. While caveolar transport, annexin A2, calcium-dependent signaling, and extracellular vesicle release have all been proposed, no unified model has emerged [33,34].

Another major gap is ENO-1 target validation by pharmacologic inhibition or antibody blockade. This issue is especially important for ENOblock, whose mechanism remains debated. Work has argued that ENOblock does not directly inhibit enolase’s catalytic activity in vitro and may interfere with certain enzymatic assays due to its optical properties [92]. Jung et al. initially reported that ENOblock directly binds enolase and inhibits its catalytic activity. They reported that ENOblock bound enolase in affinity-matrix pull down experiments, providing evidence for ENOblock-enolase binding, but did not determine the precise binding site or whether ENOblock bound to enolase’s catalytic site. To test whether ENOblock inhibited enolase enzymatic activity, they used purified enolase and measured the conversion of 2-phosphoglycerate to PEP, with the key finding that ENOblock reduced measured enolase activity to a level similar to sodium fluoride, a known enolase inhibitor. They concluded that ENOblock was the first non-substrate analog inhibitor that directly binds enolase and could be used to probe glycolytic and non-glycolytic enolase functions [78]. Satani et al. questioned whether ENOblock did inhibit enolase catalytic activity. They failed to find catalytic inhibition when using NADH-coupled and ^31^P nuclear magnetic resonance assays and showed that ENOblock’s absorbance could interfere with the spectrophotometric detection of PEP. They found that ENOblock increased baseline absorbance at 240 nm, indicating that its optical properties could interfere with the detection of PEP [92]. While these findings question ENOblock’s ability to inhibit enolase’s catalytic activity, they do not completely invalidate the biological and preclinical data reported with ENOblock, as they do not exclude the possibility that ENOblock binds to or modulate the non-glycolytic functions of ENO-1 or act through other mechanisms. Future studies should further investigate ENOblock’s target validation, non-glycolytic functions, or additional molecular targets.

Another point to consider is that most of the studies of ENO-1 have been conducted in preclinical models, including in vitro cell cultures and in vivo animal models that attempt to reproduce human inflammatory disease. While these preclinical studies are important for better understanding the underlying mechanisms of ENO-1 in inflammation and helping to establish the proof of concept with anti-ENO-1 therapeutics, they cannot completely replicate human pathophysiology, including the effects of patient comorbidities, disease heterogeneity, and the effects of current treatment guidelines. In addition, there may be species-specific differences in immune responses. Preclinical models also occasionally have incomplete confirmation of compartment-specific ENO-1 targets, which may limit translation to humans. The preclinical findings should be further validated in patient tissues, primary human cell lines, and ex vivo organoids. These translational limitations are important considerations for emerging anti-ENO-1 therapies. Although anti-ENO-1 antibodies such as HuL001 provide a strategy to target ENO-1 functions, it is currently in an early clinical translation phase, and it is still unclear which patient populations and diseases would be most appropriate for ENO-1-directed therapy [85].

## 9. Conclusion and Future Directions

In conclusion, ENO-1 has been identified as more than just a housekeeping glycolytic enzyme, but as a compartment-specific moonlighting protein that depends heavily on its subcellular location. ENO-1 has been shown to act in the cytosol, where its expression and function are altered by various stressors, and to play an active regulatory role in macrophage inflammation during sepsis. ENO-1 also contributes to inflammation via its role as a cell-surface plasminogen receptor, an extracellular innate immune signal, and in the nucleus as a transcriptional repressor. Altered ENO-1 expression has been found to converge across many different inflammatory disease states, including sepsis, acute respiratory distress syndrome, acute organ injury, hemorrhagic shock, RA, and cancer-associated inflammation in the tumor microenvironment.

The multifunctionality of ENO-1 creates several important unanswered questions. Which diseases and patient populations are the most likely to benefit from ENO-1-directed therapy, and could circulating ENO-1 levels or cell surface ENO-1 expression be used as biomarkers for patient stratification? To determine which patient population would benefit the most from ENO-1 directed therapy, prospective studies should be performed measuring circulating ENO-1, cell-surface ENO-1 expression on monocytes and neutrophils, and anti-ENO-1 autoantibodies with disease activity, severity, and response to treatment, which may guide patient selection. These studies should determine whether ENO-1 as a biomarker can correlate with a specific disease. Given the safety concerns of inhibiting a fundamental glycolytic enzyme, could a cell surface or extracellular ENO-1 be selectively targeted while preserving the needed housekeeping function of glycolytic ENO-1? Could ENO-1-directed therapies be combined with established treatment, such as antibiotics in sepsis or immune-based therapies in cancer? At least in terms of sepsis therapeutics, combination with antibiotics could certainly enhance the efficacy of ENO-1 directed therapies. What mechanisms regulate ENO-1 translocation to the cell surface and the extracellular space, and can these pathways themselves be targeted therapeutically? Ideally, studies should also determine the specific extracellular, cell-surface, or intracellular ENO-1 pathways to help guide compartment-specific therapy. Overall, future work should be based on defining the cell-type and compartment-specific roles of ENO-1 in vivo, understanding the molecular mechanisms of translocation of ENO-1 to the cell-surface or extracellularly, and which diseases are the most suitable for anti-ENO-1 therapies. As immunometabolism is essential to the inflammatory response, ENO-1 is emerging as a key molecule with relevance for therapeutic intervention and biomarker development across many immune-mediated disease states.

## Data Availability

No new data were created or analyzed in this study. Data sharing is not applicable.

## Abbreviations

The following abbreviations are used in this manuscript:

2PG	2-phosphoglycerate
ALI	Acute lung injury
ALT	Alanine aminotransferase
AST	Aspartate aminotransferase
ATP	Adenosine triphosphate
BALF	Bronchoalveolar lavage fluid
CEP-1	Citrullinated enolase-1 peptide 1
CLP	Cecal ligation and puncture
COX-2	Cyclooxygenase-2
DAMP	Damage-associated molecular pattern
ENO-1	Enolase-1
ENO-2	Enolase-2
ENO-3	Enolase-3
ERK	Extracellular signal-regulated kinase
FLS	Fibroblast-like synoviocytes
GSDMD	Gasdermin D
HIF-1α	Hypoxia-inducible factor-1 alpha
HUVECs	Human umbilical vein endothelial cells
ICAM-1	Intercellular adhesion molecule-1
IFITM2	Interferon-induced transmembrane protein 2
IFN-γ	Interferon-gamma
IL	Interleukin
IL1R2	Interleukin-1 receptor type 2
IPF	Idiopathic pulmonary fibrosis
LPS	Lipopolysaccharide
MAPK	Mitogen-activated protein kinase
mAb	Monoclonal antibody
MBP-1	Myc promoter-binding protein-1
MPO	Myeloperoxidase
NADH	Reduced nicotinamide adenine dinucleotide
NETs	Neutrophil extracellular traps
NK-κB	Nuclear factor-kappa B
NLRP3	Nucleotide-binding oligomerization domain-like receptor family pyrin domain containing 3
OXPHOS	Oxidative phosphorylation
PAR-2	Protease-activated receptor-2
PBMCs	Peripheral blood mononuclear cells
PDAC	Pancreatic ductal adenocarcinoma
PEP	Phosphoenolpyruvate
PI3K-Akt-mTOR	Phosphoinositide 3-kinase-protein kinase B-mechanistic target of rapamycin
RA	Rheumatoid arthritis
RAP1B	RAS-related protein 1B
RA-FLSs	Rheumatoid arthritis fibroblast-like synoviocytes
STIM1	Stromal interaction molecule 1
ORAI1	Calcium release-activated calcium channel protein 1
TGF-β	Transforming growth factor-beta
TLR4	Toll-like receptor 4
TME	Tumor microenvironment
TNF-α	Tumor necrosis factor-alpha
tPA	Tissue plasminogen activator
Tregs	Regulatory T cells
uPA	Urokinase-type plasminogen activator
